# Adrenal Myelolipoma Inside an Adrenal Cortical Adenoma

**DOI:** 10.7759/cureus.79563

**Published:** 2025-02-24

**Authors:** Selin Kurt, Harjot Gill, Garrison Pease

**Affiliations:** 1 Pathology, Albert Einstein College of Medicine, Montefiore Medical Center, Bronx, USA

**Keywords:** adrenal adenoma, adrenal cortical adenoma, adrenal gland, adrenal neoplasms, myelolipoma

## Abstract

A case of an incidental adrenal myelolipoma inside a non-functioning adrenal cortical adenoma is reported. A 65-year-old woman with a history of right-sided clear cell renal cell carcinoma presented for a follow-up of a left-sided adrenal mass first noted incidentally on a previous renal cell carcinoma workup. Macroscopically, it was a well-circumscribed, tan-yellow mass with gritty hemorrhage. Histopathologic evaluation showed the proliferation of clear to eosinophilic cells, filled with small vacuoles with a well-delineated lesion composed of mature adipose tissue and bone marrow elements. Also, this focus of myelolipoma revealed adenoma cells inside its borders; and interestingly, trilineage hematopoiesis was mostly observed near the adenoma-myelolipoma interfaces. To our knowledge, this is a unique report mentioning some additional features about the interfaces of these lesions. It can be suggested that the presence of adrenal cortical adenoma played the main stressor role in giving rise to myelolipoma and myelolipoma, being closer to the adenoma's vascular network, caused the different interface features.

## Introduction

Adrenal myelolipomas are uncommon benign tumors composed of mature adipose tissue and normal hematopoietic elements [1]. They are typically asymptomatic; however, larger ones may lead to mass-effect symptoms, such as abdominal discomfort or retroperitoneal bleeding, which could require surgical intervention. Rarely, patients can also present with endocrine disorders due to the hormonal activity of concurrent adrenal hyperplasia or adenoma [1-7]. 

Recently, through advanced detection methods, adrenal myelolipomas have been more commonly found as incidentalomas by computed tomography (CT) scans or magnetic resonance imaging (MRI) [3]. They are established as the second most common benign tumors in adrenal glands, ranking just after adrenocortical adenomas [4,5]. Despite being benign, these tumors hold clinical significance as they can mimic other pathologies on imaging, especially when they have simultaneous adrenal lesions like adrenocortical adenomas [3,6-8]. 

Here, we report a rare case of incidental adrenal myelolipoma within a non-functioning adrenal cortical adenoma in a patient who has a history of renal cell carcinoma. 

## Case presentation

**Figure 1:**
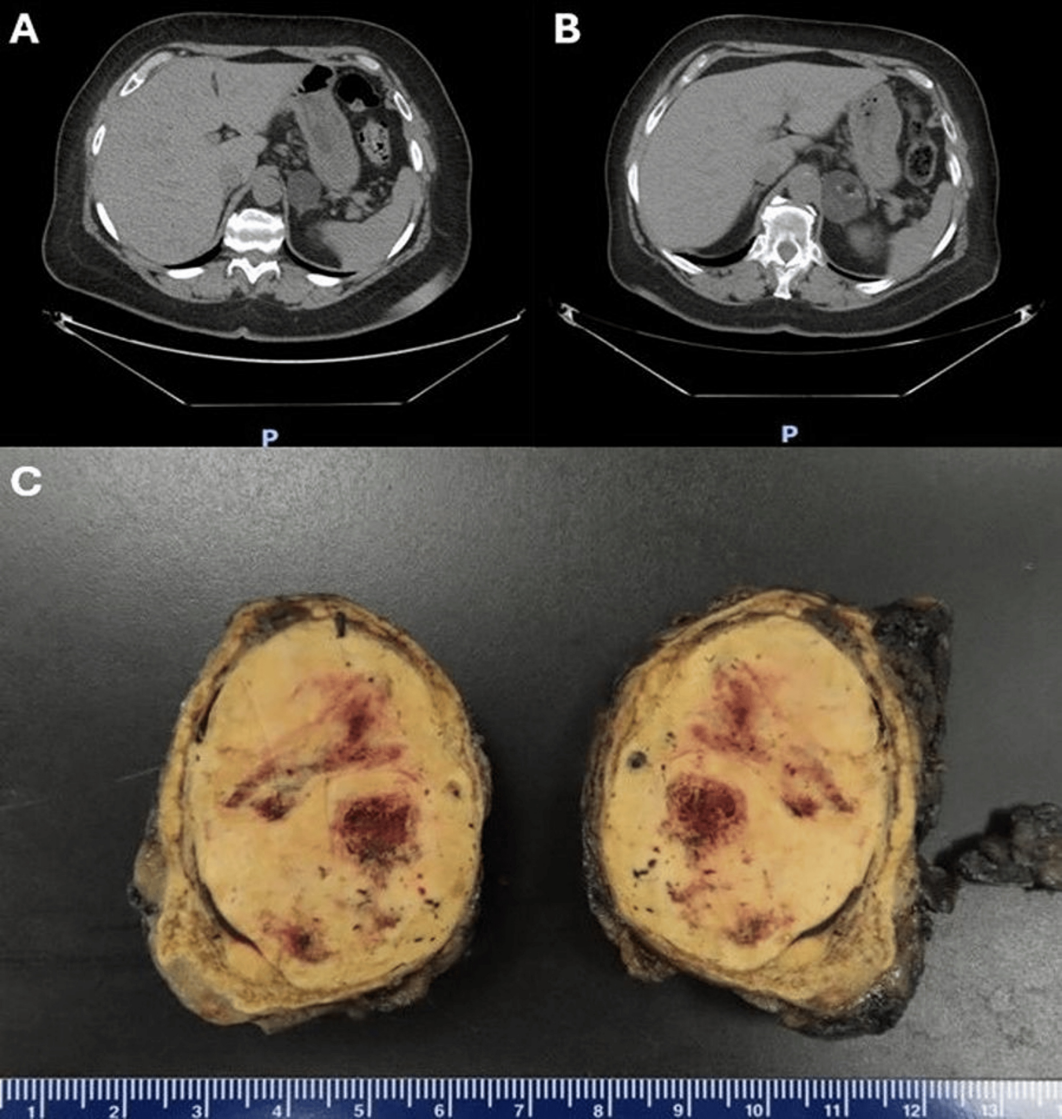
Radiology and gross pictures of the mass (A) Axial abdominal and pelvic CT scan from 2016 showing a 3.5 cm left adrenal mass, predominantly low density. (B) Follow-up axial abdominal and pelvic CT scan from 2024 demonstrating a 4.8 cm heterogeneous left adrenal mass with new macroscopic fat and calcifications, raising suspicion for a collision tumor. (C) Gross examination of the adrenalectomy specimen showing a well-circumscribed, tan-yellow, and hemorrhagic mass measuring 5.5 cm. CT: computed tomography

A 65-year-old woman with a history of hypertension, type 2 diabetes mellitus, right-sided partial nephrectomy for clear cell renal cell carcinoma, and parotidectomy for Warthin's tumor presented to the clinic for a follow-up of a 3.5 cm left-sided adrenal mass first noted in 2016 incidentally on a renal cell carcinoma workup (Figure 1A). 

During the evaluation, she reported no symptoms. A thorough physical examination and laboratory tests, including a comprehensive biochemical workup, revealed no abnormalities. Consequently, the adrenal mass was classified as hormonally inactive. 

CT scan of the abdomen and pelvis was repeated. It showed a 4.8×3.9×4.8 cm heterogeneous left adrenal mass containing a focus of macroscopic fat and tiny foci of calcification (Figure 1B). It was increased in size compared to the oldest available study from 2016 (Figure 1A), with new intralesional fat and calcifications since that time. Radiologically, the differential diagnosis of a collision tumor was proposed. 

Surgical resection was recommended because of the increased lesion size, radiologic features raising concerns for malignancy, and the difficulty of excluding a collision tumor. The patient was treated with laparoscopic left adrenalectomy. The surgery went uneventful and resulted in the removal of a solid, well-encapsulated left adrenal mass. 

Pathology examination 

Gross examination revealed that the tumor was confined to the adrenal gland and was 5.5 cm in size, well-circumscribed, tan-yellow in color, and semi-firm in consistency with a focus of hemorrhage (Figure 1C). 

**Figure 2:**
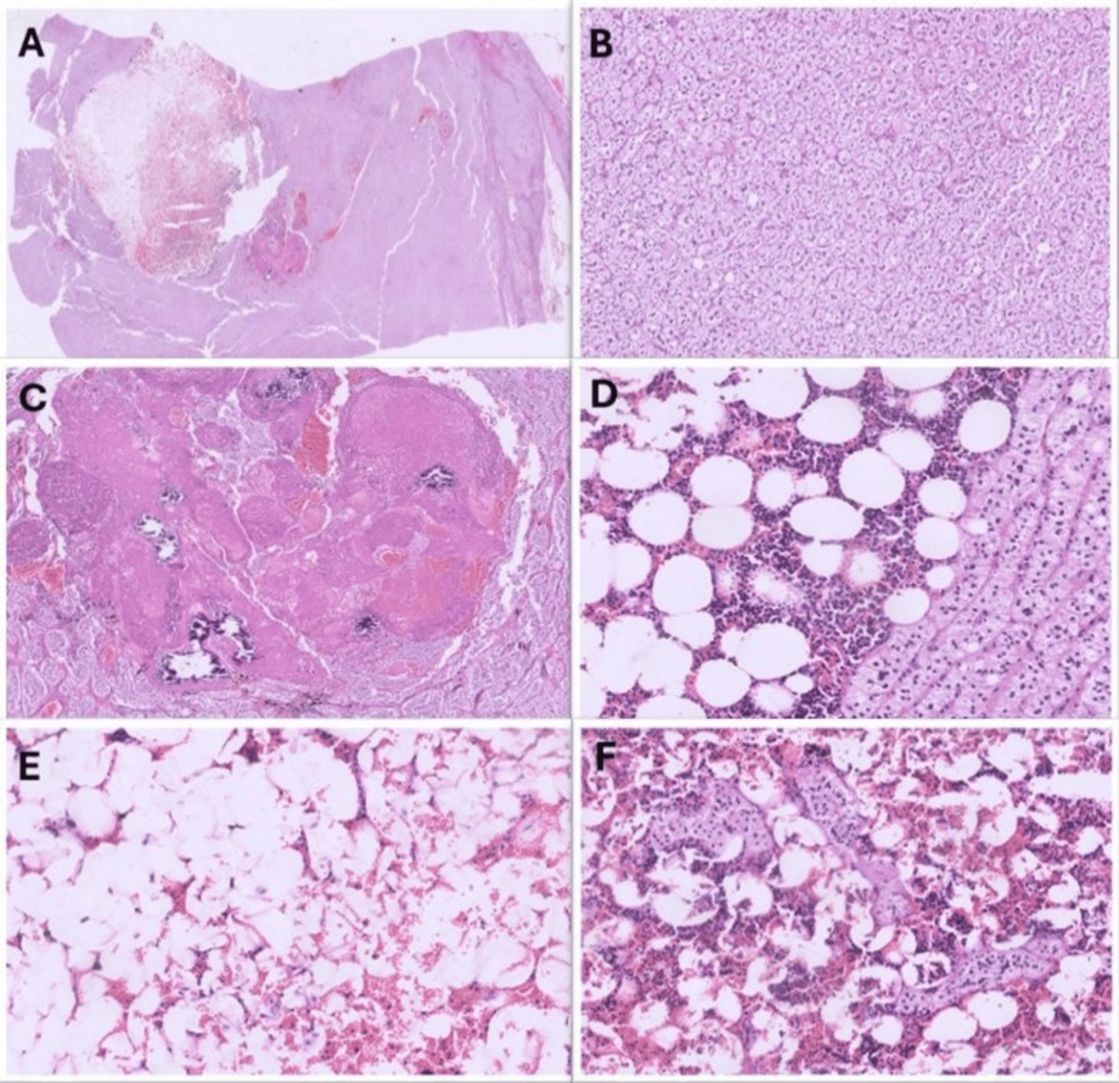
Microscopic pictures of the mass (A) Low-power view of the section from the mass showing a well-delineated hemorrhagic focus and necrosis next to it (0.5×, H&E). (B) Trabeculae of monomorphic, pale cells with lipid-rich cytoplasm (10×, H&E). (C) Necrosis and calcification foci inside the mass (2×, H&E). (D) Adipose tissue and hematopoietic elements that shows trilineage, next to the group of pale to eosinophilic cells at the well-delineated interface. (E) Mature adipose and scant hematopoietic elements from the hemorrhagic lesion (10×, H&E). F. Group of pale to eosinophilic cells inside the hemorrhagic lesion that shows lipomatous and trilinear hematopoietic elements in this area. H&E: hematoxylin and eosin

On microscopic evaluation, the tumor showed cords of clear to eosinophilic cells, filled with small vacuoles, which was diagnosed as cortical adenoma (Figure 2A, 2B). Also, inside this adenoma, a separate, well-delineated focus of mature adipose tissue and bone marrow elements was seen and interpreted as myelolipoma (Figure 2A-2F). Adenoma cells were present in this focus of myelolipoma (Figure 2F). Two focal areas of necrosis and calcification were seen (Figure 2A, 2C). No atypia or mitosis was present. The final diagnosis was myelolipoma within an adrenocortical adenoma. Due to the focal necrosis only, the total score of 1 was given according to the Modified Weiss System. 

Follow-up course

After the surgery, the patient reported fatigue and muscle cramping, but in follow-up visits, she was clinically well, and her complaints resolved, keeping with expected post-operative recovery. Since pathological examination confirmed the lesion's benign nature, no further follow-up was deemed necessary unless symptoms recur.

## Discussion

Adrenal myelolipomas are reported as isolated neoplasms or in association with other adrenal pathological conditions, such as adrenocortical adenomas, adrenocortical carcinomas, or endocrine disorders, including Conn's syndrome [3,9-11]. It has been shown that 6% of patients with adrenal myelolipomas have a concomitant functioning or non-functioning adrenal cortical adenoma [7]. Although these combinations are present, myelolipoma inside an adrenal cortical adenoma is a rare entity, and there are only a few reported cases with varying characteristics in the literature [3,9,12-15]. Our present case is the second combination case of a right-sided renal clear cell carcinoma with a left-sided adrenal myelolipoma within a cortical adenoma [12]. 

In a review of the literature, five of eight cases of myelolipoma within an adrenal cortical adenoma were in women patients. In three cases, the masses were in the right-sided adrenal, and two of them were functioning, whereas, in five left-sided adrenal masses, just one of them was functioning [3,9,12-15]. Our case differs from other cases in terms of the presence of necrosis (Figure 2A, 2C). We also observed that trilineage hematopoiesis was more prominent near the adenoma-myelolipoma interface, including in the foci of the adenoma elements inside the myelolipoma (Figure 2F). Further from the adenoma, trilineage hematopoiesis was scarce, demonstrating mostly adipose elements (Figure 2D, 2E). While we propose that the vascular network of the adenoma may play a role, this remains an observational finding without direct functional validation. Future studies like vascular mapping and molecular profiling may help clarify the mechanisms behind this distribution pattern.

The above findings cause inquiries about the nature of adrenal myelolipomas and their presence in the other adrenal masses. However, the pathogenesis of adrenal myelolipomas is still unknown. Previous studies suggested that the primary event is a metaplastic change occurring in the reticuloendothelial cells of the blood capillaries due to stimuli like necrosis, infection, or stress [6]. It is also found that adrenal myelolipomas occur predominantly on the right side, so a theory proposed by Lin et al. suggests that the right adrenal gland experiences mechanical stress from friction against the inferior border of the liver during respiration, which may contribute to the development of adrenal myelolipomas [16]. On the other hand, our review of cases showed that myelolipomas inside the cortical adenomas were mostly in the left adrenal gland. While we hypothesize that the adrenal cortical adenoma may have acted as a stressor contributing to myelolipoma formation in these cases, this remains speculative, and further evidence is required to support this theory.

The classification of myelolipomas as reactive or neoplastic remains debated. Although some studies have shown genetic alterations, such as translocations, nonrandom X-chromosome inactivation, and miRNA dysregulation, the clinical significance of these findings is still unclear. Our case supports the possibility that myelolipomas arise in response to local stimuli, but further genetic and molecular studies are needed to establish their true nature [7,15,17,18]. 

As discussed above, it is evident that more assessments of these lesions should be done carefully with the combination of clinical, biochemical, histopathologic, and molecular findings [19].

## Conclusions

A rare case of an incidental adrenal myelolipoma inside a non-functioning adrenal cortical adenoma is presented in this case report. This lesion was interpreted as a possible collision tumor radiologically. Despite the patient being asymptomatic, it was surgically removed because of the possibility of the presence of a malignant component and the growth in the lesion. Therefore, whether occurring independently or in combination with other lesions, myelolipomas remain clinically significant due to their potential to mimic malignant masses on imaging. Pathologic diagnosis of myelolipomas is mostly not challenging, but their presence with different neoplasms, like adrenal cortical adenomas, can cause difficulties, such as deciding the separate nature of the myelolipoma. Further studies are needed to accurately define the biological and pathological characteristics of these lesions.
